# Tetra­kis(μ-2-iodo­benzoato-κ^2^ *O*:*O*′)bis­[aqua­copper(II)]

**DOI:** 10.1107/S1600536812010367

**Published:** 2012-03-14

**Authors:** Ömür Aydın, Nagihan Çaylak Delibaş, Hacali Necefoğlu, Tuncer Hökelek

**Affiliations:** a Department of Chemistry, Kafkas University, 36100 Kars, Turkey; b Department of Physics, Sakarya University, 54187 Esentepe, Sakarya, Turkey; c Department of Physics, Hacettepe University, 06800 Beytepe, Ankara, Turkey

## Abstract

In the centrosymmetric binuclear title complex, [Cu_2_(C_7_H_4_IO_2_)_4_(H_2_O)_2_], the two Cu^II^ ions [Cu⋯Cu = 2.6009 (5) Å] are bridged by four 2-iodo­benzoate (IB) ligands. The four nearest O atoms around each Cu^II^ ion form a distorted square-planar arrangement, the distorted square-pyramidal coordination being completed by the O atom of the water mol­ecule at a distance of 2.1525 (16) Å. The dihedral angle between the benzene ring and the carboxyl­ate group is 25.67 (13)° in one of the independent IB ligands and 6.44 (11)° in the other. The benzene rings of the two independent IB ligands are oriented at a dihedral angle of 86.61 (7)°. In the crystal, O—H⋯O inter­actions link the mol­ecules into a two-dimensional network. π–π contacts between the benzene rings [centroid–centroid distances = 3.810 (2) and 3.838 (2) Å] may further stabilize the structure.

## Related literature

For niacin, see: Krishnamachari (1974 ▶). For *N*,*N*-diethyl­nicotinamide, see: Bigoli *et al.* (1972 ▶). For related structures, see: Speier & Fulop (1989 ▶); Usubaliev *et al.* (1980 ▶); Hökelek *et al.* (1995 ▶, 2009*a* ▶,*b* ▶,*c* ▶, 2011 ▶); Necefoğlu *et al.* (2010*a* ▶,*b* ▶).
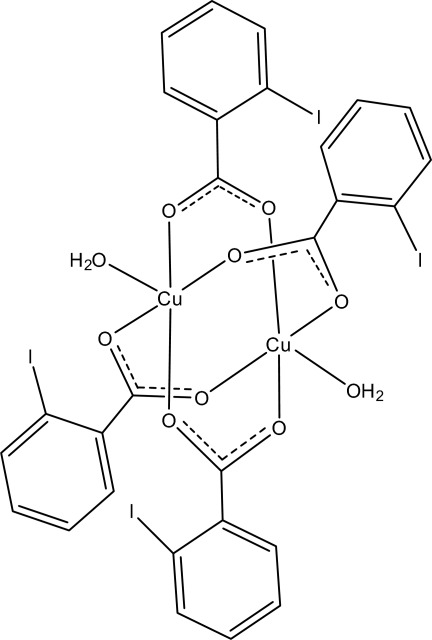


## Experimental

### 

#### Crystal data

[Cu_2_(C_7_H_4_IO_2_)_4_(H_2_O)_2_]
*M* *_r_* = 1151.14
Triclinic, 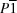

*a* = 7.3563 (2) Å
*b* = 10.7448 (3) Å
*c* = 10.9066 (3) Å
α = 83.167 (3)°
β = 72.779 (2)°
γ = 77.227 (2)°
*V* = 801.73 (4) Å^3^
*Z* = 1
Mo *K*α radiation
μ = 5.23 mm^−1^
*T* = 100 K
0.39 × 0.36 × 0.24 mm

#### Data collection

Bruker Kappa APEXII CCD area-detector diffractometer
Absorption correction: multi-scan (*SADABS*; Bruker, 2005 ▶) *T*_min_ = 0.150, *T*_max_ = 0.285
14335 measured reflections
3987 independent reflections
3818 reflections with *I* > 2σ(*I*)
*R*_int_ = 0.025

#### Refinement

*R*[*F* ^2^ > 2σ(*F* ^2^)] = 0.019
*wR*(*F* ^2^) = 0.045
*S* = 1.16
3987 reflections
207 parameters
2 restraints
H atoms treated by a mixture of independent and constrained refinement
Δρ_max_ = 0.61 e Å^−3^
Δρ_min_ = −0.66 e Å^−3^

### 

Data collection: *APEX2* (Bruker, 2007 ▶); cell refinement: *SAINT* (Bruker, 2007 ▶); data reduction: *SAINT*; program(s) used to solve structure: *SHELXS97* (Sheldrick, 2008 ▶); program(s) used to refine structure: *SHELXL97* (Sheldrick, 2008 ▶); molecular graphics: *ORTEP-3 for Windows* (Farrugia, 1997 ▶); software used to prepare material for publication: *WinGX* (Farrugia, 1999 ▶) and *PLATON* (Spek, 2009 ▶).

## Supplementary Material

Crystal structure: contains datablock(s) I, global. DOI: 10.1107/S1600536812010367/bq2345sup1.cif

Structure factors: contains datablock(s) I. DOI: 10.1107/S1600536812010367/bq2345Isup2.hkl

Additional supplementary materials:  crystallographic information; 3D view; checkCIF report

## Figures and Tables

**Table 1:** Selected bond lengths (Å)

Cu1—O1	1.9814 (16)
Cu1—O2^i^	1.9577 (16)
Cu1—O3	1.9533 (16)
Cu1—O4	1.9610 (16)

Symmetry code: (i) 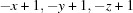.

**Table 2:** Hydrogen-bond geometry (Å, °)

*D*—H⋯*A*	*D*—H	H⋯*A*	*D*⋯*A*	*D*—H⋯*A*
O5—H51⋯O1^ii^	0.83 (3)	2.09 (3)	2.839 (2)	152 (3)
O5—H52⋯O4^ii^	0.83 (3)	2.56 (4)	3.171 (2)	132 (3)

Symmetry code: (ii) 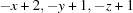.

**Fig. 1.**
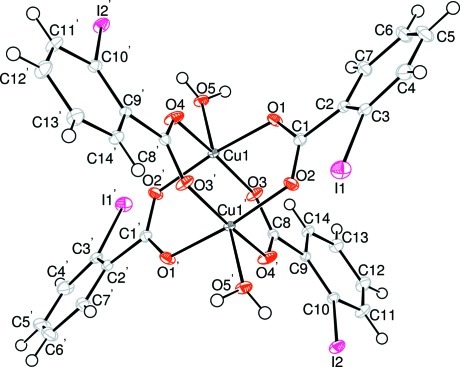
The molecular structure of the title compound with the atom-numbering scheme. Displacement ellipsoids are drawn at the 50% probability level. Primed atoms are generated by the symmetry operator:(') - x, - y, - z.

## supplementary crystallographic information

### Comment

As a part of our ongoing investigation on transition metal complexes of nicotinamide (NA), one form of niacin (Krishnamachari, 1974), and/or the nicotinic acid derivative *N*,*N*-diethylnicotinamide (DENA), an important respiratory stimulant (Bigoli *et al.*, 1972), the title compound was synthesized and its crystal structure is reported herein.

The title compound is a binuclear compound, consisting of four iodobenzoate (IB) ligands. The structures of similar complexes of the Cu^2+^, Zn^2+^ and Co^2+^ ions, [Cu(C_6_H_5_COO)_2_(C_5_H_5_N)]_2_ (Usubaliev *et al.*, 1980); [Cu(C_6_H_5_CO_2_)_2_(Py)]_2_ (Speier & Fulop, 1989); [Cu_2_(C_6_H_5_COO)_4_(C_10_H_14_N_2_O)_2_] (Hökelek *et al.*, 1995) [Cu_2_(C_8_H_7_O_2_)_4_(C_6_H_6_N_2_O)_2_] (Necefoğlu *et al.*, 2010*a*) [Zn_2_(C_11_H_14_NO_2_)_4_(C_10_H_14_N_2_O)_2_] (Hökelek *et al.*, 2009*a*); [Zn_2_(C_8_H_8_NO_2_)_4_(C_10_H_14_N_2_O)_2_].2H_2_O (Hökelek *et al.*, 2009*b*); [Zn_2_(C_9_H_10_NO_2_)_4_(C_10_H_14_N_2_O)_2_] (Hökelek *et al.*, 2009*c*); [Zn_2_(C_8_H_7_O_2_)_4_(C_10_H_14_N_2_O)_2_] (Necefoğlu *et al.*, 2010*b*) and [Co_2_(C_11_H_14_NO_2_)_4_(C_10_H_14_N_2_O)_2_] (Hökelek *et al.*, 2011) have also been determined. In these structures, the benzoate ion acts as a bidentate ligand.

The title dimeric complex, [Cu_2_(IB)_4_(H_2_O)_2_], has a centre of symmetry and two Cu^II^ atoms are surrounded by four IB groups and two water molecules. The IB groups act as bridging ligands. The Cu···Cu' distance is 2.6009 (5) Å. The average Cu-O distance is 2.0012 (16) Å (Table 1), and four O atoms of the bridging IB ligands around each Cu atom form a distorted square plane. The Cu atom lies 0.1869 (3) Å below the least-squares plane. The average O-Cu-O bond angle is 92.48 (7)°. A distorted square-pyramidal arrangement around each Cu atom is completed by the water O atom at 2.1525 (16) Å from the Cu atom (Table 1). The O5-Cu1···Cu1' angle is 176.38 (5)° and the dihedral angle between plane through Cu1, O1, O2, C1, Cu1', O1', O2', C1' and the plane through Cu1, O3, O4, C8, Cu1', O3', O4', C8' is 89.13 (6)°. The dihedral angles between the planar carboxylate groups [(O1/O2/C1) and (O3/O4/C8)] and the adjacent benzene rings A (C2-C7) and B (C9-C14) are 25.67 (13) and 6.44 (11) °, respectively, while that between rings A and B is A/B = 86.61 (7)°.

In the crystal structure, intermolecular O-H···O interactions (Table 2) link the molecules into a two-dimensional network, in which they may be effective in the stabilization of the structure. The π–π contacts between the benzene rings, Cg1—Cg1^i^ and Cg2—Cg2^ii^ [symmetry codes: (i) 1 - x, -y, 1 - z, (ii) 2 - x, 1 - y, -z, where Cg1 and Cg2 are the centroids of the rings A (C2-C7) and B (C9-C14), respectively] may further stabilize the structure, with centroid-centroid distances of 3.810 (2) and 3.838 (2) Å].

### Experimental

The title compound was prepared by the reaction of CuSO_4_.5H_2_O (1.25 g, 5 mmol) in H_2_O (100 ml) with sodium 2-iodobenzoate (2.70 g, 10 mmol) in H_2_O (50 ml). The mixture was set aside to crystallize at ambient temperature for one day, giving green single crystals.

### Refinement

Atoms H51 and H52 (for H_2_O) were located in a difference Fourier map and refined isotropically. The C-bound H-atoms were positioned geometrically with C—H = 0.95 Å, for aromatic H-atoms, and constrained to ride on their parent atoms, with *U*_iso_(H) = 1.2 × *U*_eq_(C).

### Crystal data

**Table:**

[Cu_2_(C_7_H_4_IO_2_)_4_(H_2_O)_2_]	*Z* = 1
*M**_r_* = 1151.14	*F*(000) = 538
Triclinic, *P*1	*D*_x_ = 2.384 Mg m^−^^3^
Hall symbol: -P 1	Mo *K*α radiation, λ = 0.71073 Å
*a* = 7.3563 (2) Å	Cell parameters from 9932 reflections
*b* = 10.7448 (3) Å	θ = 2.7–28.4°
*c* = 10.9066 (3) Å	µ = 5.23 mm^−^^1^
α = 83.167 (3)°	*T* = 100 K
β = 72.779 (2)°	Block, green
γ = 77.227 (2)°	0.39 × 0.36 × 0.24 mm
*V* = 801.73 (4) Å^3^	

### Data collection

**Table:**

Bruker Kappa APEXII CCD area-detector diffractometer	3987 independent reflections
Radiation source: fine-focus sealed tube	3818 reflections with *I* > 2σ(*I*)
Graphite monochromator	*R*_int_ = 0.025
φ and ω scans	θ_max_ = 28.4°, θ_min_ = 2.0°
Absorption correction: multi-scan (*SADABS*; Bruker, 2005)	*h* = −9→9
*T*_min_ = 0.150, *T*_max_ = 0.285	*k* = −14→14
14335 measured reflections	*l* = −14→14

### Refinement

**Table:**

Refinement on *F*^2^	Primary atom site location: structure-invariant direct methods
Least-squares matrix: full	Secondary atom site location: difference Fourier map
*R*[*F*^2^ > 2σ(*F*^2^)] = 0.019	Hydrogen site location: inferred from neighbouring sites
*wR*(*F*^2^) = 0.045	H atoms treated by a mixture of independent and constrained refinement
*S* = 1.16	*w* = 1/[σ^2^(*F*_o_^2^) + (0.0134*P*)^2^ + 0.9745*P*] where *P* = (*F*_o_^2^ + 2*F*_c_^2^)/3
3987 reflections	(Δ/σ)_max_ = 0.002
207 parameters	Δρ_max_ = 0.61 e Å^−^^3^
2 restraints	Δρ_min_ = −0.66 e Å^−^^3^

### Special details

**Table:**

Geometry. All esds (except the esd in the dihedral angle between two l.s. planes) are estimated using the full covariance matrix. The cell esds are taken into account individually in the estimation of esds in distances, angles and torsion angles; correlations between esds in cell parameters are only used when they are defined by crystal symmetry. An approximate (isotropic) treatment of cell esds is used for estimating esds involving l.s. planes.
Refinement. Refinement of F^2^ against ALL reflections. The weighted R-factor wR and goodness of fit S are based on F^2^, conventional R-factors R are based on F, with F set to zero for negative F^2^. The threshold expression of F^2^ > 2sigma(F^2^) is used only for calculating R-factors(gt) etc. and is not relevant to the choice of reflections for refinement. R-factors based on F^2^ are statistically about twice as large as those based on F, and R- factors based on ALL data will be even larger.

### Fractional atomic coordinates and isotropic or equivalent isotropic displacement parameters (Å^2^)

**Table:**

	*x*	*y*	*z*	*U*_iso_*/*U*_eq_	
I1	0.36263 (2)	0.882863 (16)	0.200310 (15)	0.01953 (5)	
I2	−0.20180 (2)	0.701971 (15)	0.777993 (14)	0.01579 (5)	
Cu1	0.65575 (4)	0.46820 (2)	0.53900 (2)	0.00769 (6)	
O1	0.6886 (2)	0.64727 (16)	0.49086 (16)	0.0152 (3)	
O2	0.4156 (2)	0.70018 (16)	0.43065 (16)	0.0153 (3)	
O3	0.4755 (2)	0.52102 (17)	0.70398 (15)	0.0151 (3)	
O4	0.7945 (2)	0.43067 (18)	0.35988 (15)	0.0171 (4)	
O5	0.9024 (2)	0.41164 (17)	0.61507 (15)	0.0137 (3)	
H51	1.010 (3)	0.375 (3)	0.573 (3)	0.031 (9)*	
H52	0.920 (6)	0.462 (3)	0.660 (3)	0.050 (12)*	
C1	0.5690 (3)	0.7241 (2)	0.4403 (2)	0.0111 (4)	
C2	0.6219 (3)	0.8508 (2)	0.3878 (2)	0.0118 (4)	
C3	0.5596 (3)	0.9249 (2)	0.2873 (2)	0.0137 (4)	
C4	0.6304 (4)	1.0353 (2)	0.2372 (3)	0.0204 (5)	
H4	0.5932	1.0828	0.1663	0.024*	
C5	0.7553 (4)	1.0769 (3)	0.2899 (3)	0.0247 (6)	
H5	0.8022	1.1529	0.2558	0.030*	
C6	0.8112 (4)	1.0070 (2)	0.3925 (3)	0.0222 (5)	
H6	0.8927	1.0367	0.4310	0.027*	
C7	0.7483 (3)	0.8947 (2)	0.4384 (2)	0.0162 (5)	
H7	0.7918	0.8456	0.5063	0.019*	
C8	0.2958 (3)	0.5622 (2)	0.7233 (2)	0.0102 (4)	
C9	0.1865 (3)	0.6078 (2)	0.8544 (2)	0.0095 (4)	
C10	−0.0093 (3)	0.6684 (2)	0.8928 (2)	0.0115 (4)	
C11	−0.0913 (3)	0.7150 (2)	1.0144 (2)	0.0170 (5)	
H11	−0.2233	0.7576	1.0386	0.020*	
C12	0.0184 (4)	0.6997 (3)	1.1009 (2)	0.0184 (5)	
H12	−0.0385	0.7317	1.1841	0.022*	
C13	0.2108 (4)	0.6377 (2)	1.0661 (2)	0.0160 (5)	
H13	0.2860	0.6261	1.1255	0.019*	
C14	0.2927 (3)	0.5930 (2)	0.9441 (2)	0.0124 (4)	
H14	0.4251	0.5510	0.9206	0.015*	

### Atomic displacement parameters (Å^2^)

**Table:**

	*U*^11^	*U*^22^	*U*^33^	*U*^12^	*U*^13^	*U*^23^
I1	0.01733 (8)	0.02241 (9)	0.01989 (8)	−0.00265 (6)	−0.00902 (6)	0.00232 (6)
I2	0.01043 (8)	0.01989 (8)	0.01694 (7)	0.00135 (6)	−0.00594 (6)	−0.00335 (6)
Cu1	0.00592 (12)	0.00946 (13)	0.00725 (11)	−0.00149 (9)	−0.00117 (9)	−0.00054 (9)
O1	0.0129 (8)	0.0116 (8)	0.0231 (8)	−0.0056 (6)	−0.0079 (7)	0.0048 (6)
O2	0.0119 (8)	0.0115 (8)	0.0249 (8)	−0.0044 (6)	−0.0082 (7)	0.0022 (6)
O3	0.0092 (8)	0.0237 (9)	0.0101 (7)	0.0023 (7)	−0.0018 (6)	−0.0046 (6)
O4	0.0096 (8)	0.0315 (10)	0.0090 (7)	−0.0007 (7)	−0.0018 (6)	−0.0048 (7)
O5	0.0084 (8)	0.0194 (9)	0.0134 (7)	−0.0012 (7)	−0.0037 (6)	−0.0024 (6)
C1	0.0111 (10)	0.0123 (10)	0.0082 (9)	−0.0025 (8)	0.0004 (8)	−0.0016 (8)
C2	0.0088 (10)	0.0101 (10)	0.0146 (10)	−0.0011 (8)	−0.0006 (8)	−0.0009 (8)
C3	0.0089 (10)	0.0131 (11)	0.0166 (10)	−0.0003 (8)	−0.0009 (8)	−0.0010 (8)
C4	0.0148 (12)	0.0171 (12)	0.0252 (12)	−0.0012 (10)	−0.0040 (10)	0.0069 (10)
C5	0.0194 (13)	0.0147 (12)	0.0396 (15)	−0.0089 (10)	−0.0066 (11)	0.0069 (11)
C6	0.0179 (12)	0.0147 (12)	0.0365 (14)	−0.0071 (10)	−0.0095 (11)	0.0011 (10)
C7	0.0152 (11)	0.0136 (11)	0.0197 (11)	−0.0039 (9)	−0.0048 (9)	0.0007 (9)
C8	0.0118 (10)	0.0091 (10)	0.0097 (9)	−0.0040 (8)	−0.0021 (8)	0.0002 (7)
C9	0.0092 (10)	0.0087 (10)	0.0095 (9)	−0.0029 (8)	−0.0004 (8)	0.0001 (7)
C10	0.0099 (10)	0.0119 (10)	0.0129 (10)	−0.0016 (8)	−0.0042 (8)	0.0001 (8)
C11	0.0115 (11)	0.0206 (12)	0.0146 (11)	0.0017 (9)	0.0003 (9)	−0.0041 (9)
C12	0.0168 (12)	0.0238 (13)	0.0124 (10)	−0.0005 (10)	−0.0012 (9)	−0.0068 (9)
C13	0.0161 (12)	0.0197 (12)	0.0124 (10)	−0.0026 (9)	−0.0041 (9)	−0.0027 (9)
C14	0.0104 (10)	0.0134 (11)	0.0129 (10)	−0.0006 (8)	−0.0036 (8)	−0.0006 (8)

### Geometric parameters (Å, º)

**Table:**

I1—C3	2.100 (2)	C4—H4	0.9500
I2—C10	2.102 (2)	C5—C4	1.388 (4)
Cu1—Cu1^i^	2.6009 (5)	C5—H5	0.9500
Cu1—O1	1.9814 (16)	C6—C5	1.386 (4)
Cu1—O2^i^	1.9577 (16)	C6—H6	0.9500
Cu1—O3	1.9533 (16)	C7—C6	1.374 (3)
Cu1—O4	1.9610 (16)	C7—H7	0.9500
Cu1—O5	2.1525 (16)	C8—O4^i^	1.260 (3)
O1—C1	1.272 (3)	C8—C9	1.498 (3)
O2—Cu1^i^	1.9577 (16)	C9—C10	1.403 (3)
O2—C1	1.247 (3)	C9—C14	1.397 (3)
O3—C8	1.260 (3)	C10—C11	1.388 (3)
O4—C8^i^	1.260 (3)	C11—C12	1.387 (3)
O5—H51	0.828 (18)	C11—H11	0.9500
O5—H52	0.828 (19)	C12—C13	1.384 (3)
C2—C1	1.499 (3)	C12—H12	0.9500
C2—C7	1.397 (3)	C13—C14	1.384 (3)
C3—C2	1.404 (3)	C13—H13	0.9500
C3—C4	1.389 (3)	C14—H14	0.9500
			
O1—Cu1—Cu1^i^	86.36 (5)	C5—C4—C3	120.5 (2)
O1—Cu1—O5	95.68 (7)	C5—C4—H4	119.8
O2^i^—Cu1—Cu1^i^	82.79 (5)	C4—C5—H5	120.1
O2^i^—Cu1—O1	168.98 (7)	C6—C5—C4	119.8 (2)
O2^i^—Cu1—O4	90.09 (8)	C6—C5—H5	120.1
O2^i^—Cu1—O5	95.26 (7)	C5—C6—H6	120.1
O3—Cu1—Cu1^i^	83.11 (5)	C7—C6—C5	119.7 (2)
O3—Cu1—O1	89.93 (7)	C7—C6—H6	120.1
O3—Cu1—O2^i^	90.68 (7)	C2—C7—H7	119.1
O3—Cu1—O4	168.90 (7)	C6—C7—C2	121.7 (2)
O3—Cu1—O5	93.88 (6)	C6—C7—H7	119.1
O4—Cu1—Cu1^i^	86.00 (5)	O3—C8—O4^i^	124.6 (2)
O4—Cu1—O1	87.22 (8)	O3—C8—C9	116.33 (18)
O4—Cu1—O5	97.08 (7)	O4^i^—C8—C9	119.06 (19)
O5—Cu1—Cu1^i^	176.38 (5)	C10—C9—C8	125.79 (19)
C1—O1—Cu1	119.86 (14)	C14—C9—C8	116.50 (19)
C1—O2—Cu1^i^	125.63 (15)	C14—C9—C10	117.66 (19)
C8—O3—Cu1	124.96 (14)	C9—C10—I2	125.52 (16)
C8^i^—O4—Cu1	121.09 (15)	C11—C10—I2	113.88 (16)
Cu1—O5—H51	123 (2)	C11—C10—C9	120.6 (2)
Cu1—O5—H52	117 (3)	C10—C11—H11	119.8
H51—O5—H52	108 (4)	C12—C11—C10	120.3 (2)
O1—C1—C2	116.23 (19)	C12—C11—H11	119.8
O2—C1—O1	124.6 (2)	C11—C12—H12	120.0
O2—C1—C2	119.2 (2)	C13—C12—C11	120.1 (2)
C3—C2—C1	124.2 (2)	C13—C12—H12	120.0
C7—C2—C1	117.6 (2)	C12—C13—H13	120.3
C7—C2—C3	118.1 (2)	C14—C13—C12	119.4 (2)
C2—C3—I1	125.24 (17)	C14—C13—H13	120.3
C4—C3—I1	114.73 (17)	C9—C14—H14	119.0
C4—C3—C2	120.0 (2)	C13—C14—C9	121.9 (2)
C3—C4—H4	119.8	C13—C14—H14	119.0
			
Cu1^i^—Cu1—O1—C1	−2.22 (16)	C3—C2—C7—C6	0.1 (4)
O2^i^—Cu1—O1—C1	7.9 (5)	I1—C3—C2—C1	5.4 (3)
O3—Cu1—O1—C1	−85.32 (17)	I1—C3—C2—C7	−177.62 (17)
O4—Cu1—O1—C1	83.95 (17)	C4—C3—C2—C1	−174.1 (2)
O5—Cu1—O1—C1	−179.21 (16)	C4—C3—C2—C7	2.9 (3)
Cu1^i^—Cu1—O3—C8	0.27 (18)	I1—C3—C4—C5	177.2 (2)
O1—Cu1—O3—C8	86.62 (19)	C2—C3—C4—C5	−3.3 (4)
O2^i^—Cu1—O3—C8	−82.38 (19)	C6—C5—C4—C3	0.6 (4)
O4—Cu1—O3—C8	11.6 (5)	C7—C6—C5—C4	2.3 (4)
O5—Cu1—O3—C8	−177.70 (18)	C2—C7—C6—C5	−2.7 (4)
Cu1^i^—Cu1—O4—C8^i^	−4.82 (18)	O3—C8—C9—C10	173.4 (2)
O1—Cu1—O4—C8^i^	−91.37 (18)	O3—C8—C9—C14	−3.7 (3)
O2^i^—Cu1—O4—C8^i^	77.94 (18)	O4^i^—C8—C9—C10	−5.2 (3)
O3—Cu1—O4—C8^i^	−16.0 (5)	O4^i^—C8—C9—C14	177.7 (2)
O5—Cu1—O4—C8^i^	173.25 (18)	C8—C9—C10—C11	−175.2 (2)
Cu1—O1—C1—O2	8.5 (3)	C8—C9—C10—I2	3.2 (3)
Cu1—O1—C1—C2	−170.57 (14)	C14—C9—C10—I2	−179.69 (16)
Cu1^i^—O2—C1—O1	−11.8 (3)	C14—C9—C10—C11	1.9 (3)
Cu1^i^—O2—C1—C2	167.28 (15)	C8—C9—C14—C13	176.4 (2)
Cu1—O3—C8—O4^i^	3.5 (3)	C10—C9—C14—C13	−1.0 (3)
Cu1—O3—C8—C9	−175.06 (14)	I2—C10—C11—C12	179.93 (19)
C3—C2—C1—O1	153.2 (2)	C9—C10—C11—C12	−1.5 (4)
C3—C2—C1—O2	−25.9 (3)	C10—C11—C12—C13	0.1 (4)
C7—C2—C1—O1	−23.8 (3)	C11—C12—C13—C14	0.8 (4)
C7—C2—C1—O2	157.1 (2)	C12—C13—C14—C9	−0.4 (4)
C1—C2—C7—C6	177.3 (2)		

Symmetry code: (i) −*x*+1, −*y*+1, −*z*+1.

### Hydrogen-bond geometry (Å, º)

**Table:**

*D*—H···*A*	*D*—H	H···*A*	*D*···*A*	*D*—H···*A*
O5—H51···O1^ii^	0.83 (3)	2.09 (3)	2.839 (2)	152 (3)
O5—H52···O4^ii^	0.83 (3)	2.56 (4)	3.171 (2)	132 (3)

Symmetry code: (ii) −*x*+2, −*y*+1, −*z*+1.
